# Improving the Utility of a Dynorphin Peptide Analogue Using Mannosylated Glycoliposomes

**DOI:** 10.3390/ijms22157996

**Published:** 2021-07-27

**Authors:** Jordan D. Lewicky, Nya L. Fraleigh, Alexandrine L. Martel, Thi M.-D. Nguyen, Peter W. Schiller, Leila Mousavifar, René Roy, Anh Dzung Le, Douglas Funk, Hoang-Thanh Le

**Affiliations:** 1 Health Sciences North Research Institute, 56 Walford Road, Sudbury, ON P3E 2H3, Canada; jlewicky@hsnri.ca (J.D.L.); nfraleigh@hsnri.ca (N.L.F.); amartel@hsnri.ca (A.L.M.); 2 Laboratory of Chemical Biology and Peptide Research, Montreal Clinical Research Institute, 110 Pine Avenue W, Montreal, QC H2W 1R7, Canada; mai_dung_nguyen@hotmail.com (T.M.-D.N.); peter.schiller@ircm.qc.ca (P.W.S.); 3 Department of Pharmacology and Physiology, University of Montreal, 2900 Boulevard Édouard-Montpetit, Montreal, QC H3T 1J4, Canada; 4 Glycosciences and Nanomaterial Laboratory, Department of Chemistry, Université du Québec à Montréal, P.O. Box 8888, Succ. Centre-ville, Montreal, QC H3C 3P8, Canada; leilyanmousavifar@gmail.com (L.M.); roy.rene@uqam.ca (R.R.); 5 Centre for Addiction & Mental Health, 33 Ursula Franklin Street, Toronto, ON M5S 2S1, Canada; Anh.Le@camh.ca (A.D.L.); Douglas.Funk@camh.ca (D.F.); 6 Department of Pharmacology and Toxicology, University of Toronto, 1 King’s College Circle, Toronto, ON M5S 1A8, Canada; 7 Department of Psychiatry, University of Toronto, 250 College Street, Toronto, ON M5T 1R8, Canada; 8 Medicinal Sciences Division, Northern Ontario School of Medicine, 935 Ramsey Lake Road, Sudbury, ON P3E 2C6, Canada; 9 Department of Chemistry and Biochemistry, Laurentian University, 935 Ramsey Lake Road, Sudbury, ON P3E 2C6, Canada; 10 Department of Biology, Laurentian University, 935 Ramsey Lake Road, Sudbury, ON P3E 2C6, Canada

**Keywords:** CNS therapeutic, blood–brain barrier, targeted delivery, glycoliposome, kappa opioid receptor antagonist, addiction, dynantin, peptide, dopamine, neurotransmitter

## Abstract

Peptide therapeutics offer numerous advantages in the treatment of diseases and disorders of the central nervous system (CNS). However, they are not without limitations, especially in terms of their pharmacokinetics where their metabolic lability and low blood–brain barrier penetration hinder their application. Targeted nanoparticle delivery systems are being tapped for their ability to improve the delivery of therapeutics into the brain non-invasively. We have developed a family of mannosylated glycoliposome delivery systems for targeted drug delivery applications. Herein, we demonstrate via in vivo distribution studies the potential of these glycoliposomes to improve the utility of CNS active therapeutics using dynantin, a potent and selective dynorphin peptide analogue antagonist of the kappa opioid receptor (KOR). Glycoliposomal entrapment protected dynantin against known rapid metabolic degradation and ultimately improved brain levels of the peptide by approximately 3–3.5-fold. Moreover, we linked this improved brain delivery with improved KOR antagonist activity by way of an approximately 30–40% positive modulation of striatal dopamine levels 20 min after intranasal administration. Overall, the results clearly highlight the potential of our glycoliposomes as a targeted delivery system for therapeutic agents of the CNS.

## 1. Introduction

Peptides have long been considered to hold great potential as pharmacotherapeutic agents, generally having favorable properties for this application that include high target specificity and potency, with minimal potential for immunogenic reaction or interactions with other drugs [1,2]. Peptides do not accumulate in tissues and are effectively metabolized by endogenous enzymes into non-toxic amino acid metabolites [3]. However, peptides are not without inherent limitations, which include their poor physical and metabolic stability, and low permeation of biological membranes due to their high molecular weight and hydrophilicity [3,4]. Despite these limitations, the number of peptides that have gained regulatory approval as pharmaceutics continues to grow [5].

Peptides play an important role in the CNS where they are involved in, mediate, or are themselves affected by many of the physiological functions of the brain, spinal cord, and associated nerves [6]. It is not surprising, therefore, that peptides have long been sought for the treatment of diseases and disorders of the CNS, where their potency, specificity and low toxicity are advantageous [7]. In addition to their inherent metabolic instability, the major obstacle and limitation in the design of CNS-active peptide therapeutics has been their low permeability across the blood–brain barrier (BBB) [8,9]. While the function of this barrier is the maintenance of brain homeostasis and protection from potentially damaging agents, its highly selective nature excludes all large-molecule therapeutics such as peptides and the majority of small-molecule drugs [10].

There has been extensive interest in the use of nanoparticle (NP) delivery systems as a non-invasive approach to improve both the metabolic stability and delivery of therapeutics of all sizes across the BBB without having an effect on their activity [11,12,13,14,15]. Liposomal NPs are an ideal carrier modality for therapeutics destined for the CNS due to their biocompatibility, biodegradability, and their unique physicochemical properties that allow for the incorporation of both hydrophilic and lipophilic payloads [16]. The transcytotic activity of receptors expressed at the BBB has been exploited via the active targeting of NPs whose surfaces are decorated with the respective ligands [17]. A wide array of receptors has been investigated for active CNS targeting of NPs and the list continues to grow [15]. While the transmembrane glycoprotein mannose receptor has been used considerably to target therapeutic-loaded NPs on both immune cells and various types of cancer cells [18,19,20,21,22,23,24,25], the CNS targeting potential of the mannose ligand is only beginning to be appreciated. The mannose receptor is expressed on the macrophages and microglia of the BBB [26] and the use of mannosylated liposomes has been shown to improve the CNS levels of a variety of structurally diverse therapeutics [27,28,29,30].

**Figure 1:**
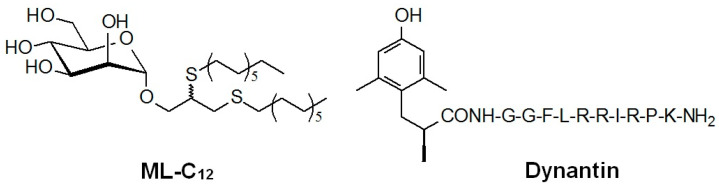
Structure of ML-C_12_ and dynantin.

We developed a mannosylated liposomal NP delivery system in collaboration with the laboratory of Dr. Roy, comprising a C_12_-alkyl-mannopyranoside (ML-C_12_, Figure 1) member of a library of novel amphiphilic neoglycolipids that self-assemble in water to form monodisperse glycoliposomes [31]. These glycoliposomes are capable of entrapping and protecting different peptide-based therapeutics from degradation in plasma ex vivo [32]. We also showed that the ML-C_12_ glycoliposome system (hereafter referred to as DS1) improved the delivery of dynantin (Figure 1), a dynorphin peptide analogue, into the CNS of mice in vivo when administered intranasally [33]. Intranasal (IN) administration has been used previously for peptides to minimize systemic delivery and improve uptake into the CNS [34,35,36]. Dynantin is being developed in collaboration by our team as a peptide KOR antagonist [33,37]. KOR signaling is an integral component of the brain reward function (reviewed in [38]). Endogenous dynorphin-like KOR agonist peptides inhibit the release of dopamine in the dorsal and ventral striatum, thus playing a critical role in the negative feedback regulation of the dopamine release induced by drugs of abuse [38,39,40,41]. Potent and selective KOR antagonists such as dynantin [37] block the activity of endogenous dynorphin, ultimately resulting in increased dopamine levels in the striatum [39,42] that are associated with a unique combination of both antidepressant and antianxiogenic effects that are believed that they could have a transformative impact on the treatment of drug addiction and its associated withdrawal [43,44,45,46,47,48,49]. Clinical studies with prototypical non-peptide KOR antagonists [50,51,52,53] have identified several serious issues linked with complex pharmacokinetic and pharmacodynamic properties, including extended durations of action, which have ultimately impeded the potential benefits of these agents in humans [54,55,56]. In this regard, peptide KOR antagonists, such as dynantin, which offer many of the aforementioned advantages over non-peptide structures, continue to be investigated [57]. However, the poor BBB penetration and metabolic lability of dynantin have ultimately hampered its clinical development [32,52].

**Figure 2:**
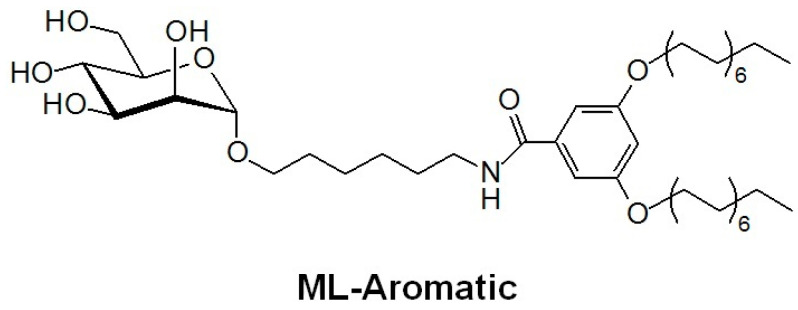
Structure of ML-Aromatic.

In this work, we extend on the CNS targeting potential of our mannosylated glycoliposomes by demonstrating their ability to improve the delivery, activity, and thus utility of dynantin as a peptide KOR antagonist. We present a novel application of an aromatic-alkyl mannopyranoside (ML-Aromatic, Figure 2) that incorporates an increased hydrophilic character in the glycolipid (synthesis modified from [58]). We assess and compare the respective glycoliposome delivery system generated from ML-Aromatic (hereafter referred to as DS2) to DS1 with respect to its ability to entrap, protect, and deliver the dynantin peptide to the brains of mice using RP-HPLC. For the first time we demonstrate the KOR antagonist activity of dynantin by way of LC/MS analysis of dopamine modulation in vitro using homogenates of fresh mouse striatal tissue. Most importantly, we link the improved CNS distribution of the peptide with its ability to positively modulate dopamine levels in the striatum of mice in vivo. Overall, the results clearly demonstrate that the mannosylated glycoliposomes hold great promise as targeted delivery systems for peptide-based CNS therapeutics.

## 2. Results and Discussion

### 2.1. Entrapment and Plasma Stability

**Figure 3:**
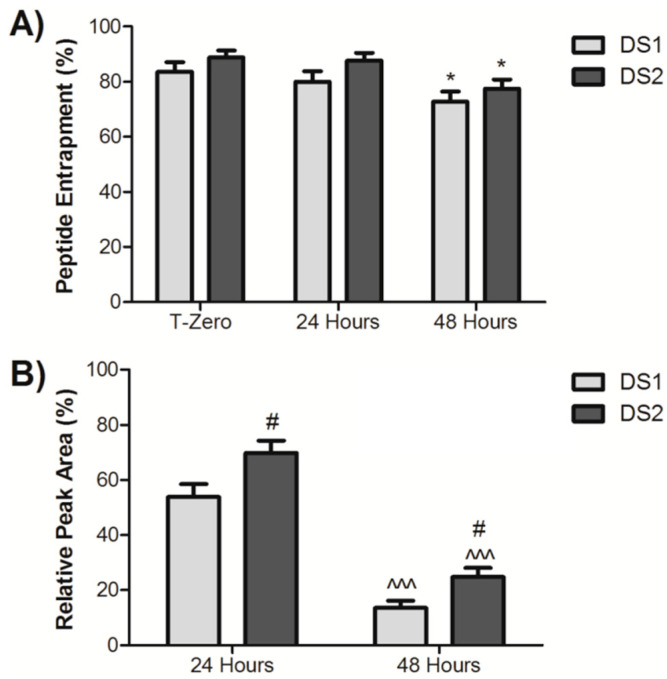
Glycoliposomal entrapment and plasma stability of dynantin. Dynantin was combined with DS1 or DS2 in water and the glycoliposome solutions were left at room temperature. Similar combinations of the peptide and delivery systems were incubated in human plasma at 37 °C. The degree of peptide entrapment (**A**) or the levels or peptide remaining (**B**) at various time points were analyzed by RP-HPLC in the presence of 0.1% trifluoroacetic acid and detected by absorbance at 210 nm. Entrapment results are represented as the percentage of entrapped peptide relative the amount determined in control samples devoid of the delivery systems. Stability results were calculated relative to the quantities determined at time point zero. All data are shown as the average ± SEM of three separate experiments. * *p* < 0.05 as compared to T-zero; ^^^ *p* < 0.001 as compared to the respective formulation at 24 h; # *p* < 0.05 as compared to the DS1 formulation at the respective time point.

Initially, the ability of DS1 and DS2 to entrap and protect dynantin from proteolytic degradation in human plasma ex vivo was compared (Figure 3A). The mannosylated glycoliposomes were comprised of the respective glycolipid and cholesterol, which we previously demonstrated improved particle physicochemical properties and reduced the amount of the glycolipid used in particle formation [33]. Each delivery system was formulated at an optimal 5:2 ratio of the glycolipid: cholesterol by weight, which corresponded to an approximately 5:3 and 5:4 molar ratio for ML-C_12_ and ML-Aromatic, respectively. Both delivery systems provided a high degree of initial entrapment (84% ± 4% for DS1 and 88% ± 3% for DS2) that was virtually maintained after 24 h (80% ± 4% for DS1 and 87% ± 3% for DS2). Only after 48 h did peptide entrapment decrease (73 % ± 4% for DS1 and 78% ± 3% for DS2). While entrapment levels with DS2 trended higher than with DS1 at each time point, the differences did not achieve statistical significance. Human plasma stability analyses (Figure 3B) demonstrated a significant ability of the delivery systems to protect the peptide from the rapid and complete degradation that we previously determined to occur in as quickly as 12 h [32]. Significant levels of dynantin remained after both 24 and 48 h of incubation, with DS2 (70% ± 4% after 24 h and 25% ± 3% after 48 h) providing a significantly higher level of protection than DS1 (54% ± 5% after 24 h and 14% ± 3% after 48 h) at either time point. These results suggest that particles formed with the aromatic glycolipid are more resistant to degradation.

### 2.2. In Vitro Dopamine Modulation

**Figure 4:**
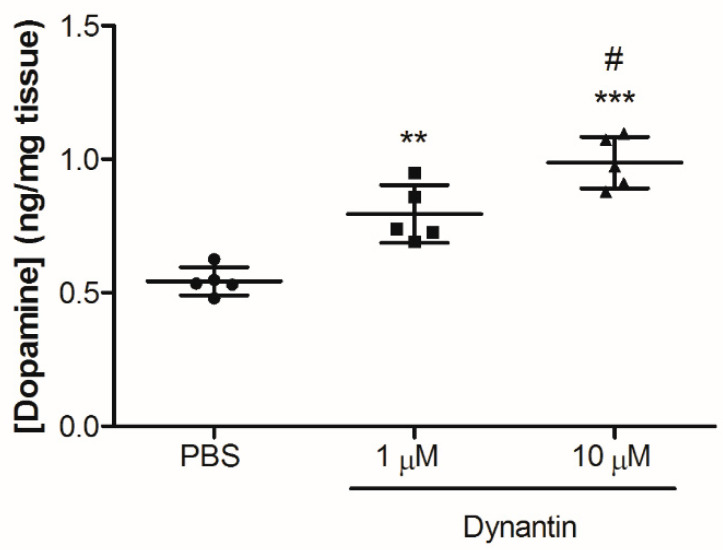
Dynantin in vitro dopamine modulation. Striatal tissue was collected from the brains of sacrificed mice (*n* = 15), gently homogenized and then treated with either PBS (60 µL, *n* = 5) or dynantin in PBS at a concentration of either 1 µM (60 µL, *n* = 5) or 10 µM (60 µL, *n* = 5) for 90 min at 4 °C in the dark. Dopamine was extracted and its levels analyzed by LC/MS in the presence of 0.1% formic acid. Data are shown as the average ± SD of the 5–6 mice in each group. *** *p* < 0.001 and ** *p* < 0.01 as compared to the PBS treatment group, # *p* < 0.05 as compared to the 1 µM dynantin treatment group.

The ability of dynantin to antagonize the KOR and modulate dopamine levels was next investigated in vitro using homogenates of the fresh mouse brain striatum (Figure 4) and LC/MS, a highly sensitive analytical technique that can be combined with a variety of different extraction methods for the analysis of neurotransmitter levels in rodent brain tissue [59,60,61,62,63]. The gently homogenized tissue was treated with either PBS or dynantin at 1 and 10 µM concentrations for 90 min, after which dopamine levels were analyzed. To date, the KOR antagonist properties of dynantin have only been demonstrated in vitro in ligand displacement binding affinity assays and in the functional guinea pig ileum assay [37]. For the first time, the results of our in vitro study demonstrate that dynantin is able to increase striatal dopamine levels by binding the KOR and blocking the activity of endogenous dynorphin. Dopamine levels were significantly elevated in the 10 µM dynantin treatment group (0.98 ± 0.09 ng/mg) as compared to the group treated with the peptide at the 1 µM concentration (0.78 ± 0.10 ng/mg). Both dynantin concentrations resulted in dopamine levels that were significantly elevated with respect to the PBS control group (0.54 ± 0.07 ng/mg). Importantly, the levels of striatal dopamine measured in the PBS treated control group are within the normal range of baseline levels reported in the literature for mice [61,62,63], indicating that the dissection and extraction processes did not have any significant effect.

### 2.3. In Vivo Dynantin Distribution and Dopamine Modulation

**Figure 5:**
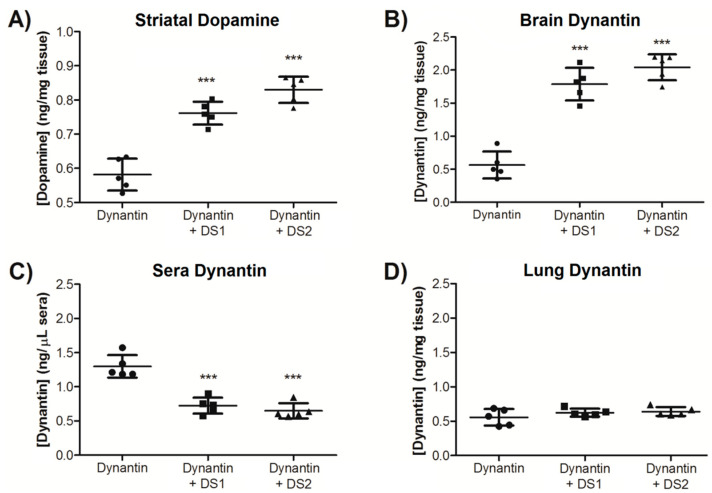
Dynantin in vivo distribution and dopamine modulation. Dynantin (2.0 µg total dose) was administered intranasally to female BALB/c mice (5 per group) either alone or entrapped with DS1 or DS2 (1:5 ratio of peptide: particles). The mice were euthanized 20 min after administration and the lung, brain, and blood (sera) were collected. Brains were immediately dissected on ice to obtain striatum tissue from which dopamine was extracted and its levels analyzed by LC/MS in the presence of 0.1% formic acid (**A**). Dynantin was extracted from the remaining brain tissue (**B**), sera (**C**), and lungs (**D**) after lyophilisation and levels of dynantin quantified by RP-HPLC in the presence of 0.1% trifluoroacetic acid and detection by absorbance. Data are shown as the average ± SD of the 5 mice in each group. *** *p* < 0.001 as compared to the peptide devoid of a delivery system.

The ability of the glycoliposome delivery systems to improve both the delivery and activity of dynantin in the brain was studied in an in vivo mouse model. Female BALB/c mice (5 per group) were intranasally administered dynantin (2.0 µg dose) alone or entrapped in either DS1 or DS2. Mice were sacrificed 20 min later and blood (sera), lungs and brains were collected. The duration of these studies was chosen partly based on a previous report in which the effects of a novel fast-acting non-peptide KOR antagonist were observed as short as 30 min after its systemic administration [64]. Striatum tissue was immediately removed and neurotransmitter levels analyzed by LC/MS. Overall, the dopamine modulatory effects observed in vitro are supported by the results of these in vivo distribution studies. Dopamine levels (Figure 5A) were significantly higher in mice that were administered the peptide in either DS1 (0.76 ± 0.04 ng/mg) or DS2 (0.83 ± 0.05 ng/mg) than in those administered the peptide alone (0.58 ± 0.05 ng/mg). Dynantin levels in the remaining brain tissue (Figure 5B), sera (Figure 5C), and lungs (Figure 5D) were analyzed by RP-HPLC using our established methods [33]. While there were detectable levels of dynantin in the brains of the mice that were administered the peptide alone (0.56 ± 0.20 ng/mg), significantly higher levels were found in the brains of the mice treated with DS1 (1.79 ± 0.25 ng/mg) or DS2 (2.04 ± 0.20 ng/mg) entrapped peptide. The opposite trend was observed in the sera, where there were significantly lower levels of the peptide when entrapped in either DS1 (0.73 ± 0.12 ng/µL) or DS2 (0.65 ± 0.11 ng/µL) than with the peptide administered alone (1.30 ± 0.16 ng/µL). Similar levels of the peptide were found in the lungs of the three different groups of mice (0.56 ± 0.12 ng/mg for dynantin alone, 0.62 ± 0.08 ng/mg for DS1, and 0.64 ± 0.06 ng/mg for DS2), although the results with the delivery systems were of lower variability. Similar trends can be seen when the data is examined in terms of the percentage of the total dynantin dose recovered from the organ and sera samples (Table 1), where DS1 and DS2 improve the overall distribution of the peptide into the brain by approximately 3.0 and 3.5 times, respectively. Total dynantin recoveries were on par with our previous studies [33] when factoring in the reduced amount of tissue analyzed.

IN administration can lead to a direct transfer of peptide and protein therapeutics to the brain, effectively bypassing the blood–brain barrier by utilizing the neuronal distribution pathways in the olfactory epithelium [64]. Depositing the therapeutic on the difficult to target olfactory region of the nasal cavity is critical to achieving the direct nose-to-brain delivery [65,66]. The fact that some dynantin does reach the brain when the peptide is administered alone suggests that a certain degree of direct nose-to-brain transfer is occurring with the IN administration. It is reasonable to predict that far less of the peptide would reach the brain with systemic administration of the peptide alone due to its known pharmacokinetic limitations [32,52]. It is not possible to discern if direct nose-to brain delivery is occurring when the peptide is entrapped in the delivery systems and, in fact, the data indicates that there is significant systemic absorption of the peptide occurring, both when used alone and when in the particles. Nonetheless, the results clearly demonstrate the brain targeting ability offered by the mannosylation of the glycoliposomes, in that there is significantly less peptide remaining in the systemic circulation. Similar levels of the peptide were found in the lungs of all three groups, although the reduced variability observed with the delivery systems may be a product of the particles’ ability to prevent degradation and clearance. Taken together, the results clearly highlight the ability of DS1 and DS2 to improve both the delivery and activity of dynantin in the brain, where the resulting higher levels of the peptide translate into significant positive modulations of striatal dopamine levels. While not statistically different, the data is trending towards DS2 offering improved brain delivery of the peptide. Although the in vivo IN administration method we used is effective in the present study to demonstrate the utility of the delivery systems, it is far from optimized and it may be suggested that with improved methods of administration, more robust effects of DS2 in terms of its targeting ability could be achieved. On that note, the fact that there is a lower molar ratio of glycolipid in DS2 than in DS1 suggests that factors other than the targeting ability of the mannose residue are involved, that which we suspect involves the balance of hydrophilic/hydrophobic character in the particles. Ultimately, this will require further investigation to elucidate and will be presented in a separate publication.

The results of this study demonstrate a significant potential for the mannosylated glycoliposome systems DS1 and DS2 to improve the utility of the KOR antagonist peptide dynantin and overcome the pharmacokinetic limitations that have hindered its development. The ability of the peptide to modulate striatal dopamine levels has now been confirmed. Future studies involving behavioral assessments in animals will investigate these dynantin delivery systems for their ability to aid in the treatment of addiction and withdrawal from drugs of abuse. We also aim to extend the application of these delivery systems to other peptide- and non-peptide-based CNS therapeutics and explore the potential of other mannosylated liposomes having a different balance of hydrophilic/hydrophobic constituents to elucidate the role this plays in blood–brain barrier penetration by the particles.

## 3. Materials and Methods

### 3.1. Reagents

Dynantin was prepared according to previously published methods [37] and was stored as a lyophilized powder at −20 °C. RP-HPLC indicated the purity of the peptide to be ≥98%. A stock solution of the peptide was generated in ddH_2_O (5 µg/µL), aliquoted, and stored at −80 °C.

The preparation of ML-C_12_ (DS1) and ML-Aromatic (DS2) followed previously published methods [31,58]. Details of the design and properties of ML-Aromatic are to be presented in a separate publication that is currently in preparation. Purity of the glycolipids was determined to be ≥95% as indicated by thin-layer chromatography and nuclear magnetic resonance spectroscopy. Stock solutions (50 µg/µL) of the glycolipids or cholesterol (≥99%, Sigma Aldrich, Fairlawn, NJ, USA) were prepared by dissolving the compounds in tert-Butanol (>99.5%, Alfa Aesar, Ottawa, ON, Canada), and stored at −20 °C.

### 3.2. Human Plasma Collection

Blood was collected from healthy volunteers in blood collection tubes containing EDTA (BD Vacutainer, Mississauga, ON, Canada). The tubes were centrifuged at 900× *g* and 20 °C for 10 min with decreased deceleration to obtain plasma, which was aliquoted and stored at −80 °C. All protocols were approved by the Research Ethics Board (Protocol # 18-061) and the Biosafety Committee at Health Sciences North Research Institute.

### 3.3. HPLC Conditions

All analyses were performed using a Shimadzu Prominence series HPLC system (Shimadzu Corporation, Kyoto, Japan), equipped with a LC-20AB binary pump, SIL-20A HT autosampler, CTO-20AC temperature-controlled column oven, SPD-M20A photodiode array detector, and CBM-20A communications bus. All equipment was controlled by Shimadzu Lab Solutions Lite software version 5.71 SP2. For separation, an Ultra C18 column, 3 μm, 50 mm × 4.6 mm (RESTEK Corporation, Bellefonte, PA, USA) was used. Dynantin samples were analyzed at 35 °C and a constant solvent flow rate of 0.7 mL/min using a binary gradient (Table 2). Solvent A consisted of a 25% solution of acetonitrile (HPLC grade, Fisher Scientific, Fairlawn, NJ, USA) in ddH_2_O (0.2 μm filtered) and solvent B consisted of acetonitrile with each solvent containing 0.1% trifluoroacetic acid (*v*/*v*, protein sequencing grade, Sigma Aldrich, Fairlawn, NJ, USA).

### 3.4. LC/MS Conditions

A modified version of a previously reported method was used [67]. Analyses were performed using an Acquity Class H UPLC system with an Acquity QDa detector (Waters Limited, Mississauga, ON, Canada). Separations took place on an Acquity UPLC BEH C18 column, 1.7 µm, 100 mm × 2.1 mm(Waters Limited, Mississauga, ON, Canada), and were achieved using a binary gradient elution (Table 3) of water and acetonitrile (both with 0.1% formic acid, *v*/*v*) and a uniform flow rate of 0.4 mL/min. QDa detector settings were optimized for dopamine in terms of cone voltage (5 V), capillary voltage (+0.8 V), probe temperature (600 °C), and source temperature (120 °C).

### 3.5. Entrapment and Plasma Stability

Glycoliposomal entrapment was investigated by combining the stock dynantin solution (12 µL, 60 µg) with DS1 (5:3 molar ratio of ML-C_12_: cholesterol) or DS2 (5:4 molar ratio of ML-Aromatic: cholesterol) delivery systems (both in tert-butanol, 10 µL total addition) in ddH_2_O to a final volume of 100 µL and a final ratio of dynantin: particles of 1:5 (*w*/*w*). Three sets of samples were each set up in triplicate, with the mixtures gently vortexed for 5 min. For one replicate of each sample, solids were immediately pelleted by centrifugation at 14,000 rpm and 20 °C for 10 min, and supernatants carefully removed for analysis of the levels of non-entrapped peptide that remained by RP-HPLC (10 µL injections, in duplicate). The other sample sets were left at room temperature for 24 or 48 h before the non-entrapped peptide levels were determined via the same method. The degree of peptide entrapment is represented as the percentage of entrapped peptide relative to the amount determined in respective control samples comprising the peptide and tert-butanol devoid of any glycolipid and cholesterol.

Dynantin stability in combination with DS1 or DS2 was investigated using a modified version of the above noted procedure. The stock peptide solution (12 µL, 60 µg) was first combined with the glycolipids and cholesterol (both in tert-butanol, 10 µL total addition), and then thoroughly mixed before the addition of thawed human plasma (128 µL). The final ratio of dynantin: particles was 1:5 (*w*/*w*). A control sample was also prepared by combining the peptide with tert-butanol alone (10 µL) and plasma (128 µL). Samples were incubated at 37 °C in a heating mantle (VWR Scientific, Mississauga, ON, Canada) for varying lengths of time before being stored at −80 °C. For HPLC analysis, samples were thawed, thoroughly mixed, diluted in MeOH (1/10) to destroy liposome particles, and solids pelleted by centrifugation at 10,000 rpm and 20 °C for 10 min. The supernatants were carefully removed for analysis (10 µL injections, in duplicate). Stability is represented as the percentage of peptide remaining relative to the amount determined at T-zero.

### 3.6. Animals and Husbandry

Female BALB/c and C57BL/6 mice were purchased from Charles River (QC, Canada) at an age of 6–8 weeks and were housed in Innocage^®^ mouse cages at the Animal Care Facility at Laurentian University. Mice were provided specialized feed for rodents and the water used was provided in Aquavive^®^ acidified mouse water bottles (250 mL volume). Both food and water were available ad libitum. The animal room was maintained at a temperature of 21 ± 2 °C and a relative humidity of 55% ± 5%. These parameters were recorded daily in addition to maintaining 12-h light and dark cycles. Mice were randomly placed into groups of 5, and were 28 weeks of age at the time of the experiments. All protocols were approved by the Animal Care Committee at Laurentian University and the Biosafety Committee at Health Sciences North Research Institute (Protocol # 6009941).

### 3.7. In Vitro Dopamine Modulation

Male C57BL/6 mice (*n* = 15) were sacrificed by CO_2_ asphyxiation and cervical dislocation. Brains were immediately collected and dissected on ice ventral side-up by cutting along the midpoint of the anterior hypothalamic nucleus to obtain the approximate 1/3 section of brain constituting the striatum [68,69]. This tissue was gently homogenized on ice using a pellet mixer (VWR, Radnor, PA, USA) and then treated with either PBS (60 µL, *n* = 5) or dynantin in PBS at a concentration of either 1 µM (60 µL, *n* = 5) or 10 µM (60 µL, *n* = 5). Samples were left for 90 min at 4 °C in the dark before being further homogenization and ice-cold absolute ethanol containing formic acid (0.1% *v*/*v*, 240 µL) and 100 ng of a deuterated dopamine-D2 internal standard was added to the samples. The mixture was vigorously vortexed and then left on ice in the dark for 30 min before solids were pelleted at 10,000 rpm and 4 °C for 10 min. Supernatants (200 µL) were carefully removed, replaced with an equal volume of acidified ethanol, and the mixture vigorously vortexed and placed on ice for a further 30 min. Solids were again pelleted and the combined supernatants were allowed to evaporate to dryness at room temperature in the dark overnight. Remainders were reconstituted in ddH_2_O with formic acid (0.1% *v*/*v*, 100 µL) with any solids pelleted by centrifugation at 10,000 rpm and 4 °C for 5 min. The amount of dopamine extracted was analyzed via LC/MS (5 µL injections) and quantified using the dopamine: dopamine-D2 peak area ratio and a standard curve that was generated using increasing concentrations of dopamine in the presence of a set concentration of the dopamine-D2 internal standard. Dopamine levels are represented as a concentration normalized to the weight of each tissue sample.

### 3.8. In Vivo Dynantin Distribution and Dopamine Modulation

Female BALB/C mice (5 per group) under isoflurane anesthesia (SomnoSuite, Kent Scientific, Torrington, CT, USA) were administered dynantin (2.0 µg total dose) intranasally (10 µL, 5 µL per nare dropwise by a micropipette) either alone in PBS or glycoliposomally entrapped in either DS1 or DS2 (1:5 ratio of peptide: particles, *w*/*w*). Mice were sacrificed after 20 min under excess isoflurane via cardiac exsanguination and cutting of the diaphragm. In an effort to minimize the number of experimental animals required, systemic administration was not investigated due to the known pharmacokinetic limitations of dynantin [32,52].

For each mouse, blood was placed in sera tubes, which were spun at 10,000 rpm for 5 min and sera collected. Lungs were removed from each animal and immediately stored on ice. Brains were immediately dissected and dopamine was extracted from striatal tissue according to the procedure outlined above. The amount of dopamine extracted was analyzed via LC/MS (5 µL injections) and quantified using the dopamine: dopamine-D2 peak area ratio and a standard curve that was generated using increasing concentrations of dopamine in the presence of a set concentration of the dopamine-D2 internal standard. Dopamine levels are represented as a concentration normalized to the weight of each tissue sample. The remaining brain tissue, sera, and lungs were kept frozen at −80 °C for extraction and analysis of dynantin levels according to our previously published methods [33]. Dynantin levels are represented as both a total recovery and a concentration normalized to the weight or volume of each sample.

### 3.9. Statistical Analyses

Statistical analyses in the form of either a one-way ANOVA with a Tukey HSD or Mann–Whitney *t*-test were performed using Graph Pad Prism 5. The criterion for significance was *p* < 0.05.

## Figures and Tables

**Table 1:** Dynantin in vivo distribution represented as the percentage of total dose recovered from organ and sera samples.

	Brain (%)	Sera (%)	Lung (%)
Dynantin	8.15 ± 3.06	21.66 ± 3.99	4.93 ± 0.89
Dynantin + DS1	24.89 ± 4.39	14.03 ± 3.02	5.05 ± 0.98
Dynantin + DS2	28.03 ± 3.51	11.68 ± 1.18	5.50 ± 0.53

Data shown are average ± SD of the 5 mice in each group.

**Table 2:** Solvent gradient program for the analysis of dynantin entrapment, plasma stability, and in vivo distribution.

Time (min)	Solvent	
	A (%)	B (%)
0	100	0
15	40	60
18	20	80
26	20	80
30	100	0
40	100	0

Solvent A: 25% acetonitrile in water and Solvent B: acetonitrile, both with 0.1% trifluoroacetic acid (*v*/*v*). Samples were analyzed at a constant flowrate of 0.7 mL/min.

**Table 3:** The solvent gradient program for the analysis of dopamine levels.

Time (min)	Solvent	
	A (%)	B (%)
0	100	0
15	40	60
18	20	80
26	20	80
30	100	0
40	100	0

Solvent A: water and Solvent B: acetonitrile, both with 0.1% formic acid (*v*/*v*). Samples were analyzed at a constant flowrate of 0.4 mL/min.

## Data Availability

All data is contained within the manuscript.
